# Multidrug-resistant phenotype and isolation of a Novel SHV- beta-Lactamase variant in a clinical isolate of *Enterobacter cloacae*

**DOI:** 10.1186/s12929-015-0131-5

**Published:** 2015-04-11

**Authors:** Amel Bourouis, Mouhamed Ben moussa, Omrane Belhadj

**Affiliations:** Address: Laboratory of Biochemistry and Biotechnology, Faculty of Sciences of Tunis, 2092 El Manar II, Tunisia; Addresses: Laboratory of Microbiology, Military Hospital of Tunis, 1089 Monfleury, Tunisia

**Keywords:** Bacterial resistance, *Enterobacter cloacae*, ESBL, SHV-128, Mutations

## Abstract

**Background:**

ESBL-producing bacteria are a clinical problem in the management of diseases caused by these pathogens. Worldwide, systemic infections with BL enzymes are evolving by mutations from classical *bla* genes in an intensified manner and they continue to be transferred across species.

**Results:**

*E.cloacae* BF1417 isolate and its transconjugants gave positive results with the DDST, suggesting the presence of ESBL. Sequence analysis revealed a *bla*_SHV-ESBL_-type gene that differs from the gene encoding SHV-1 by five point mutations resulting in three amino acid substitutions in the coding region: C123R, I282T and L286P. This novel SHV-type enzyme was designated SHV-128. The conjugation tests and plasmid characterization showed that the *bla*_SHV-128_ is located on a conjugative plasmid IncFII type. Expression studies demonstrated that the above mutations participated in drug resistance, hydrolysis of extended spectrum β-lactam and the change of the isoelectric point of the protein.

**Conclusion:**

These findings underscore the diversity by which antibiotic resistance can arise and the evolutionary potential of the clinically important ESBL enzymes. In addition, this study highlights the need for systematic surveillance of ESBL-mediated resistance as well as in clinical areas and communities.

## Background

In Gram-negative bacteria, ESBL-mediated resistance is emerging worldwide and is mainly due to the mobilization of Ambler class A β-lactamase, the largest structural ⁄ evolutionary group [1,2]. Most ESBLs are variants of the classical TEM-1 and SHV-1 ß-lactamases, with one or more amino acid substitutions that confer resistance to broad-spectrum cephalosporins and aztreonam. These changes alter the catalytic site allowing the hydrolysis of oxyimino cephalosporins and monobactams [3,4].

SHV β-lactamases are prevalent in Gram-negative bacteria. These enzymes were originally reported in *Klebsiella pneumoniae* clinical isolate and they exhibits an overall preference for hydrolysis of sulfhydryl in cephalosporin drug (hence the SHV name) [5,6]. SHV-1 can hydrolyse penicillin and cephalosporins but not expanded-spectrum antibiotics such as oxyimino cephalosporins and monobactams. Point mutations in the SHV-1 gene were the first to be reported, and are frequently associated with several mobilization events in *bla*_SHV_ gene resulting in new β-lactamase variants [7]. At present, more than 150 SHV variants have been identified and novel ESBLs continue to be reported at an alarming rate (http://www.lahey.org/studies/). Rapidly, genes encoding these enzymes have been mutated and transferred to other Gram-negative bacteria including *Enterobacter cloacae* that is commonly found in hospitals and causes a wide range of infections, such as lower respiratory tract infections, urinary tract infections and meningitis [8]. This microorganism is the most commonly isolated member of the *Enterobacteriaceae* that possess a chromosomally encoded AmpC β-lactamase that plays an important role in resistance to antibiotics [9]. However, several reports have demonstrated that these species can acquire and express genes encoding extended-spectrum β-lactamase [10].

Until today, β-lactamase enzymes with an extended spectrum activity against the majority of β-lactams evolve at an alarming rate and new β-lactamases that are transferred among species on plasmids with multiple resistance factors are also being described continuously. In this study, we report a phenotypic and molecular characterization of a novel SHV-type β-lactamase SHV-128 in a multidrug-resistant *E.cloacae* strain.

## Methods

### Bacterial strains

*E. cloacae* BF1417 was recovered during an epidemiological study at the Military Hospital of Tunis, Tunisia. This strain was isolated from a stool culture of a 75-year-old man hospitalized for renal failure in the intensive care unit at the Military hospital of Tunis. The studied strain was selected on the basis of its multidrug-resistance phenotype (MDR) and it was identified using MALDI-TOF MS system, the VITEK 2 (bioMérieux, La Balme-les-Grottes, France) and the API 20 E system (bioMérieux, Marcy l’Etoile, France). *E. coli* DH5α competent cells were used as host for cloning and for the transformation experiments. Streptomycin-resistant *E. coli* HB101 was used as a recipient for conjugation tests.

### Resistance transfer and plasmid characterization

The transferability of ESBLs genes between the clinical isolate and the recipient was performed by conjugation experiments using the filter-mating procedure [11]. Transconjugants growing on selection plates were subjected to DDST to confirm the resistance transfer and the presence of the ESBL phenotype. Plasmid DNA of the clinical isolate and its transconjugants were extracted with a plasmid extraction kit Promega Midi Plasmid Prep according to the manufacturer’s instructions. For determination of plasmid size, the plasmid DNAs from the transconjugants and the clinical isolate were subjected to electrophoresis on 0.7% agarose gel. Plasmid incompatibility groups were determined by PCR-based replicon typing according to Carattoli et al. [12].

### Antimicrobial susceptibility testing and ESBL detection

The antimicrobial susceptibility of *E. cloacae* BF1417 to β-lactams, fluoroquinolones, phénicol, aminoglycosides and other drugs was performed on Mueller–Hinton (MH) agar plates by the standard disk diffusion procedure as described previously. Minimum inhibitory antibiotics concentrations were determined by the serial dilution method and results were interpreted according to the Clinical and Laboratory Standards Institute guidelines [13]. ESBL phenotype was confirmed by the double disk synergy test (DDST) in presence of cloxacillin at 250 μg/ml in Mueller–Hinton agar (Biorad, Marnes-la- Coquette, France).

### Analytical isoelectric focusing (IEF)

The β-lactamase contents of the clinical strain and its transconjugants were analyzed by isoelectric focusing on a pH-3 to 10 ampholine polyacrylamide (Bio-rad®, France) gel containing starch 0.5% at a voltage of 100 to 300 in a 111 Mini IEF Cell (Bio-Rad®, France). β-lactamases with known pIs were used as standards: TEM- 1(pI 5.4),TEM-2 (pI 5.6), TEM-3 (pI 6.3) and SHV- 1(pI 7.6) [14].

### β-lactamase essay and IC_50_ determination

*E. coli* DH10B/PBF1417 was grown overnight at 37°C in Trypto-Casein Soy broth (TSB) (Diagnostics, Pasteur, France) supplemented with cefotaxim, 20 μg/ml. The cells were harvested by centrifugation and washed once in 25 mM potassium-sodium phosphate buffer (pH 7) and resuspended in 1 ml of the same buffer. For preparation of cell free extract, the cells were ruptured by ultrasonic treatment in a UP 400 S sonicator at 4°C. Cell debris was removed by centrifugation at 10,000 rpm for 10 min in a Hettich centrifuge R32 Rotor. The supernatant was loaded onto a Sephadex G-75 column (95 by 2 cm; Pharmacia, Sweden) equilibrated with the same buffer. Eluted fractions were collected and tested spectrophotometrically for β-lactamase activity with 50 μM cephaloridine as the substrate at 255 nm. Active fractions were pooled, dialyzed against 25 mM Tris–HCl buffer, and then applied to a polyacrylamide slab gel electrophoresis under non-denaturing conditions as previously described [15]. After incubation with cefotaxime 20 μg/ml, β-lactamase activity was identified as a clear band appeared in the gel background. The detected band was then cut and eluted in 1 ml of the phosphate buffer (0.25 M; pH 7.4) at 4°C under moderate agitation overnight. The purity of enzymes was estimated by using sodium dodecyl sulfate-polyacrylamide gel electrophoresis [16]. Hydrolysis of the substrates was monitored by following the absorbance variations as described previously using a CARY 50 Bio UV-visible spectrophotometer connected to a microcomputer. For the partially purified enzyme, relative hydrolysis rate (Vmax) was determined graphically using Linweaver-Burk representation [17]. For determination of inhibitor effects, rates of hydrolysis of 1 mM cephalothin were determined in the presence of various concentrations of clavulanic acid and sulbactam. EDTA was used for 1 mM as concentration. In these experiments, the proteic extract was preincubated with the inhibitors for 10 min before the addition of cephalothin. The inhibitory concentration that allowed the reduction of β-lactamases activities of 50% was graphically fixed as previously described [18].

### PCR amplification and DNA sequencing

Detection of gene sequences coding for β-lactamase enzymes was performed with the plasmid DNA as template using primers specific for *bla*_TEM_, *bla*_SHV_, *bla*_CTX-M_, *bla*_OXA_ and _AmpC_ (Table 1). Positive sample was re-amplified using Pfu DNA polymerase (Promega, USA). The amplifications were performed on 100 μl samples containing 5 μl DNA, 0.2 nmol of each primers, 20 nmol deoxynucleoside triphosphate (dNTP), 3 U *Pfu* DNA polymerase) and 10 μl 10X buffer (20 mM MgSO4), and dimethyl sulfoxide (7%). PCR mapping of the genetic environment surrounding the *bla*_SHV_ gene was performed with primers specific for insertion sequence IS26 (primers IS26-1 and IS26-2) combined with forward and reverse primers for *bla*_SHV_ gene as previously described [19]. The experiment was performed on a DNA thermal cycle (AB Applied Biosystem 2720). The PCR products were separated in 1, 2% agarose gel and visualised with UV. Amplified DNA fragments were sequenced on both strands and the nucleotide sequences and their deduced amino acid sequences were compared with those included in the GenBank database using BLAST software available online (www.ncbi.nlm.nih.gov/BLAST).

**Table 1:** **Sequences of the primers used to detect β-lactamases genes**

**PCR target**	**Primer name**	**Primer sequence 5′ → 3′**	**Annealing T°**	**Ampliquons size**
CTX-M	CTX-M-A	CGCTTTGCGATGTGCAG	54	539
	CTX-M-B	ACCGCGATATCGTTGGT		
SHV	SHV-1 F	ATGCGTTATATTCGCCTGTGTAT	54	868
	SHV-1R	TTAGCGTTGCCAGTGCTCGATCAG		
TEM	TEM-A2	GTATCCGCTCATGAGACAAT	54	932
	TEM-ext	GTATATATGAGTAAACTTGGTCTG		
OXA	OXA-1 F	CACAATACATATCAACTTCGC	54	793
	OXA-1R	GTGTGTTTAGAATGGTGATCGC		

### Cloning of SHV gene

The *bla*_SHV_ PCR product was purified using microcolumns of the Microspin Sephacryl S-400 purification system (Amersham Biosciences). Purified DNA were ligated into pGEM-T easy PCR cloning vector and transformed into a competent cell of *E. coli* DH5α according to the manufacturer’s instructions (Promega, WI). Positive colonies were selected on LB agar plates supplemented with ampicillin (100 μg/ml)®. Plasmids were isolated using the Rapid Plasmid miniprep system and digested with EcoRI restriction enzyme (New England Biolabs) to confirm the presence of the insert. The insert sequences were performed on both strands by using forward and reverse primers: UM11F (5′-CACCTTGCCGACGCAATGAC-3′) and UM11 R (5′-TTAGCGTTGCCAGTGCTCG -3′), an automated fluorescent method based on dye terminator chemistry (AmpliTaq DNA polymerase FS Dye Terminator Cycle Sequencing Ready Reaction Kit; Applied Biosystems) and ABI Prism 3100 automated sequencer (Applied Biosystems, USA). A similarity search of the sequence was carried out using the BLAST program available at the NCBI BLAST homepage (www.ncbi.nlm.nih.gov/blast/).

### Nucleotide sequence accession number

The nucleotide sequence of the *bla*_SHV-128_ gene has been submitted to the EMBL-GenBank database and has been assigned accession number GU932590.

## Results

The clinical strain of *E. cloacae* BF1417 included in this study was isolated in the ICU of a Military hospital in Tunisia during 2009. Antimicrobial tests showed that the isolate was resistant to all β-lactams tested except imipenem. Resistance to other drugs, including chloramphenicol, Tetracyclin, aminoglucosides and quinolones, was observed. By filter mating, all the above resistance determinants except cefoxitin, chloramphenicol and Tetracyclin were transferred to *E.coli* HB101 and were carried on IncFII type plasmid of about 100-kb. The transconjugants expressed high resistance to broad-spectrum beta-lactams and gave positive results with the DDST, indicating the production of ESBL. Resistance to other antimicrobial agents was also transferred with the plasmid suggesting their presence in a same genetic context. The MICs of various antibiotics tested for donors, transconjugants, and *E.coli* HB101 are summarized in Table 2. MICs values revealed similar resistance profiles for most β-lactams in transconjugants and the clinical strain except the resistance to cefoxitin. When clavulanic acid was combined with cefotaxime and cefdazidime, a reduction in the MICs of these drugs was observed (Table 2). Interestingly, the quinolone resistance phenotype of the transconjugants was identical to this of the donor strain except for nalidixic acid. Indeed, the transconjugants exhibited a reduction in the CMIs from 256 μg/ml to 32 μg/ml which could be due to other factors such as chromosomal gene mutations Such as alterations in the targets of quinolones, and decreased accumulation inside the bacteria due to impermeability of the membrane and/or an overexpression of efflux pump systems [19].

**Table 2:** **Minimum inhibitory concentrations (MICs) of various antimicrobial agents obtained for the clinical isolate of** ***E.cloacae*** **and transconjugants**

**Antibiotics**	**Minimum inhibitory concentration (μg/ml)**		
	***E.cloacae*** **BF1417**	***E.coli*** **HB101**	**HB101x pBF1417**
Ampicillin	>512	4	>512
Ticarcillin	>256	2	>256
Cefoxitin	>512	2	4
Ceftazidime	128	1	128
Ceftazidime + ACL	4	ND	4
Cefotaxime	64	1	64
Cefotaxime + ACL	4	ND	4
Cefpirome	8	2	8
Aztreonam	256	1	256
Nalidixic acid	256	1	32
Ciprofloxacin	32	1	16
Tetracyclin	>512	2	2
Cloramphenicol	>256	2	2
Imipinem	<2	0,25	<2

**Figure 1:**
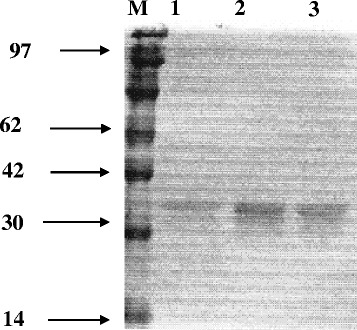
SDS-PAGE gel analysis of SHV-128 from *Escherichia coli* HB101. Lane M: molecular mass standards; lane 1: before Sephadex G-75 column, lane 2: after dialysis of active fraction, lane 3: after polyacrylamide gel analysis.

Analytical isoelectric focusing of crude extract of *E.cloacae* BxF1417 and its transconjugants revealed the presence of an identical β-lactamase band with a pI value greater than 8.6. This activity was revealed with cefotaxime as substrates, which confirms that this was an ESBL.The purity was verified by SDS-PAGE (Figure 1), relative hydrolysis rates of this enzyme for benzylpenicillin, cefotaxime, ceftazidime, and aztreonam are shown in Table 3. The substrate profile of SHV-128 was compared with those of SHV-12 and SHV-2, the first described ESBL. Compared with SHV-12, SHV-128 showed an increasing hydrolysis rate for cefotaxime and, in particular, aztreonam. The hydrolysis rate of ceftazidime was similar to those of SHV-12. However, the Vm value of the SHV-128 enzyme was lower for benzylpenicillin than those of ceftazidime and cefotaxime. Such behavior illustrates well the extended-spectrum activity of SHV-128 enzyme. Inhibition studies showed that SHV-128 was strongly inhibited by clavulanic acid, with IC50 = 4.2 μM.

**Table 3:** **Substrate profiles of SHV-128 compared to those of SHV- 2 and SHV-12 enzymes**

	**Relative Vmax**		
	**SHV-2**	**SHV-12**	**SHV-128**
Penzylpenicillin	100	100	76
Cephaloridine	123	214	ND
Ceftazidime	<1	16	17
Cefotaxime	27	42	56
Aztreonam	11	110	140

**Figure 2:**
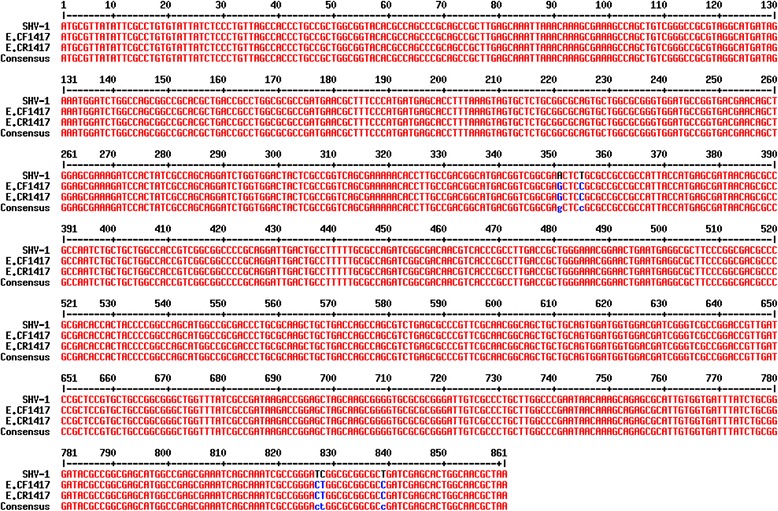
Nucleotides sequence alignment of the *bla*_SHV-128_ gene from *E.cloacae* BF 1417 with the published *bla*_SHV-1_ variant.

Polymerase chain reaction (PCR) amplification did not yield any products except for the *bla*_SHV_ gene for both, clinical isolate and transconjugants. The PCR products were purified and cloned into pGEM-T Easy cloning vector and transformed into *E. coli* DH5α. The plasmid, pGEM-128, was extracted and the sequence of the PCR-generated insert was determined on both strands. Sequence data of SHV-128 revealed an ORF of 861 pb encoding a protein product with 286 amino acid residues. Compared with the reported sequences of *bla*_SHV-1_from GenBank, the resulting sequence revealed a novel SHV-1 variant that was identical to *bla*SHV-1 except for five point mutations, A(351)G, T(355)C, T(827)C, C(828)T and T(8229) C, which was designated SHV-128 (Figure 2). Analysis of the SHV-128 and SHV-1 amino acid sequences revealed 3 amino acid substitutions in the coding region (cystein to arginin at position 123, isoleucine to threonine at position 282 and leucine to proline at position 286). PCR mapping and sequencing revealed that the *bla*_SHV-128_ gene, identified in the clinical isolate ant transconjugants, was enclosed by insertion sequence (IS) IS26 which were detected both upstream and downstream of the reported gene. Thus, the plasmid location of *bla*_SHV-128_ could facilitate its spread especially since this gene is enclosed by insertion sequence (IS) IS26 and associated with other resistant genes.

## Discussion

The present study report the phenotypic and molecular characterization of a novel extended-spectrum SHV-type β-lactamase, designated here as SHV-128. The amino acid sequence of SHV-128 differs from the amino acid sequence of SHV-1 by three amino acid substitutions: cystein for arginin at position 123, isoleucine for threonine at position 282 and leucine for proline at position 286. To our knowledge, these substitutions are observed for the first time in a natural mutant SHV-type β-lactamase (http://www.lahey.org/studies/). According to the phenotypic analysis, the resulting new enzyme induced a resistance phenotype compatible with that of an ESBL. Indeed, the susceptibility pattern showed that the enzyme caused resistance to C3G such as ceftazidime and cefotaxime and is inhibited by the β-lactamase inhibitor clavulanic acid. Furthermore, the clinical isolate and the transconjugants gave positive results with the DDST, indicating the production of ESBL. SHV-128 was located on a conjugative plasmid IncFII type of about 100-kb. The presence of resistance genes on plasmids and transposable elements allows the genes to be transferred to distantly related bacteria by conjugation, transduction, or transformation [20-23]. In fact, several studies have been conducted to evaluate the effect of promoter mutation or replacement on the ESBL expression, especially those delivered by insertion sequences IS which the case here [24].

Generally, substitutions at positions 240 and 238 seem to be especially critical for ESBL activity and occur in the vast majority of SHV-type ESBL. Indeed, previous experiments on SHV β-lactamases have reported that G238S and E240K mutations are involved in conferring resistance to C3G [25-27]. On the basis of its amino acid sequence, SHV-128 includes these residus and contains the ^70^SXXK^73^ tetrad, characteristic of β-lactamases possessing a serine active site. Two structural motifs characteristic of class A β-lactamases, were also found to be present in this novel variant: SDN at position 130–132 and KTG at position 234–236 [28]. Also, analysis of SHV-128 structure model (results not cited) showed that C123R mutation is not far from the SDN element that span over active site residues. According to previous studies [29], the arginine residu can induce changes in the electrostatic interactions in an additional manner, which might be a factor in enhancing the stability of the protein. The change of the pI and the extended spectrum of the enzyme could be attributed to the three natural mutations reported here. However, in order to analyze the role of these substitutions, a site-directed mutagenesis analysis should be done. Furthermore, expression and purification of each mutant will be required for better characterization of the SHV-128 enzyme and to specify the effect of each mutation on the active site of the enzyme and its substrate profile. The results obtained in the present study suggest that continuous mutation has led to the occurrence of novel *bla*_SHV_ ESBL gene variants. On the other hand, the presence of the insertion sequences IS suggests that resistance associated with SHV production can contribute to the dissemination of these emerging genes into other nosocomial pathogens.

## Conclusion

In conclusion, the present study explored the molecular basis of antibiotic resistance in a clinical isolate originating from Tunisian hospitals. The data revealed a novel SHV-type β-lactamase, SHV-128. This protein possessed the conserved class A β-lactamase motifs and hydrolyzed oxyimino-cephalosporins as well as aztreonam, conferring resistance to these agents. *bla*_SHV-128_ was bounded by IS26, which facilitated its acquisition and its transfer among species in the hospital. These finding highlights the genetic plasticity of the SVH-type. β-lactamases and the remarkable adaptability of *Enterobacteriaceae* species to selective antibiotic pressure.
